# Imbalances between Matrix Metalloproteinases (MMPs) and Tissue Inhibitor of Metalloproteinases (TIMPs) in Maternal Serum during Preterm Labor

**DOI:** 10.1371/journal.pone.0049042

**Published:** 2012-11-08

**Authors:** Inge Tency, Hans Verstraelen, Ivo Kroes, Gabriële Holtappels, Bruno Verhasselt, Mario Vaneechoutte, Rita Verhelst, Marleen Temmerman

**Affiliations:** 1 Department of Obstetrics and Gynecology, Faculty of Medicine and Health Sciences, Ghent University, Ghent, Belgium; 2 Upper Airways Research Laboratory, Department of Oto-rhino-laryngology, Faculty of Medicine and Health Sciences, Ghent University, Ghent, Belgium; 3 Department of Clinical Chemistry, Microbiology, and Immunology, Faculty of Medicine and Health Sciences, Ghent University, Ghent, Belgium; 4 International Centre for Reproductive Health, Department of Obstetrics and Gynecology, Faculty of Medicine and Health Sciences, Ghent University, Ghent, Belgium; John Hunter Hospital, Australia

## Abstract

**Background:**

Matrix metalloproteinases (MMPs) are involved in remodeling of the extracellular matrix (ECM) during pregnancy and parturition. Aberrant ECM degradation by MMPs or an imbalance between MMPs and their tissue inhibitors (TIMPs) have been implicated in the pathogenesis of preterm labor, however few studies have investigated MMPs or TIMPs in maternal serum. Therefore, the purpose of this study was to determine serum concentrations of MMP-3, MMP-9 and all four TIMPs as well as MMP:TIMP ratios during term and preterm labor.

**Methods:**

A case control study with 166 singleton pregnancies, divided into four groups: (1) women with preterm birth, delivering before 34 weeks (*PTB*); (2) gestational age (*GA) matched controls*, not in preterm labor; (3) women *at term in labor* and (4) *at term not in labor*. MMP and TIMP concentrations were measured using Luminex technology.

**Results:**

MMP-9 and TIMP-4 concentrations were higher in women with *PTB* vs. *GA matched controls* (resp. p = 0.01 and p<0.001). An increase in MMP-9:TIMP-1 and MMP-9:TIMP-2 ratio was observed in women with *PTB* compared to *GA matched controls* (resp. p = 0.02 and p<0.001) as well as compared to women *at term in labor* (resp. p = 0.006 and p<0.001). Multiple regression results with groups recoded as three key covariates showed significantly higher MMP-9 concentrations, higher MMP-9:TIMP-1 and MMP-9:TIMP-2 ratios and lower TIMP-1 and -2 concentrations for preterm labor. Significantly higher MMP-9 and TIMP-4 concentrations and MMP-9:TIMP-2 ratios were observed for labor.

**Conclusions:**

Serum MMP-9:TIMP-1 and MMP-9:TIMP-2 balances are tilting in favor of gelatinolysis during preterm labor. TIMP-1 and -2 concentrations were lower in preterm gestation, irrespective of labor, while TIMP-4 concentrations were raised in labor. These observations suggest that aberrant serum expression of MMP:TIMP ratios and TIMPs reflect pregnancy and labor status, providing a far less invasive method to determine enzymes essential in ECM remodeling during pregnancy and parturition.

## Introduction

Matrix metalloproteinases (MMPs) are proteolytic, zinc-dependent enzymes[1]–[6] capable of degrading extracellular matrix (ECM) components, including collagen [1], [4], [5], [7], [8]. The human MMP family currently consists of 26 members [1] and is classified according to substrate specificity into collagenases, gelatinases, stromelysines, matrilysins, membrane type-MMPs and other MMPs [1], [6], [7], [9]. More specifically, MMP-9, also known as gelatinase B, plays a role in the remodeling of collagenous ECM [1] and cleaves collagen type IV, the major basement membrane component, collagen type V and elastin [1], [2]. MMP-3 or stromelysin-1 degrades a wide range of ECM proteins and participates in proMMP activation [1], [6]. Their activity is regulated by tissue inhibitors of metalloproteinases (TIMPs) of which four have been identified.[1]–[4], [6], [10]. Inhibition of MMP activity occurs in a 1∶1 stoichiometric relationship [1], [7], [8], [11]. The balance between collagenolysis and its inhibition is critical during ECM remodeling [1], [6]. An imbalanced MMP:TIMP ratio has been involved in various medical conditions in humans including cancer, rheumatoid arthritis, osteoarthritis, endometriosis and vascular diseases [1], [7], [8].

Human pregnancy is characterized by a steady remodeling of the collagenous ECM in order to adapt fetal membranes and cervix to uterine and fetal growth as gestation progresses [4], [12]. MMPs play also a crucial role in birth-related events, including cervical ripening and dilatation and membrane weakening and rupture [2], [4]. Some MMPs (e.g. MMP-1, MMP-2 and MMP-3) are constitutively expressed during gestation, while the production of others (e.g. MMP-9) are induced by active labor [2], [3], [13], [14]. Aberrant ECM degradation by MMPs has been documented during pregnancy complications including preterm birth. Preterm birth (PTB), defined as a delivery before 37 completed weeks gestation, is a multifactorial syndrome in which intrauterine infection (IUI) is one of the most important mechanisms involved [15], [16]. IUI trigger MMP production via inflammatory mediators [17]. Activation of the MMP cascade causes ECM degradation, predisposing membrane rupture and cervix ripening [2], [12], [18].

A number of studies have shown that IUI[19]–[22], spontaneous rupture of the membranes [11], [21]–[27] and parturition[21]–[24], [26], [28] either term or preterm are associated with elevated MMP-9 concentrations in amniotic fluid, but few studies have investigated the involvement of MMP-3 in labor and parturition. Increased MMP-3 levels were found in amniotic fluid during term as well as preterm parturition [13], [29] and in cases of IUI [29], [30].

A fully functional TIMP network has been demonstrated in fetal membranes, decidua and placenta, irrespective of labor status[31]–[33]. The majority of studies focused only on TIMP-1 and TIMP-2. TIMP-1 concentrations in amniotic fluid were increased in the presence of IUI [21], [23] and in patients with rupture of the membranes either term or preterm [11], [23], but not in those with spontaneous labor [11], [24]. In contrast, TIMP-2 levels were decreased in women with IUI, rupture of the membranes and spontaneous labor [11], [27], [34]. Furthermore, it has been shown that amniotic fluid levels of TIMP-1 decrease with advancing gestational age [24], [26] while those of TIMP-2 do increase [34].

We hypothesized that aberrant MMP expressions at local level implicated in ECM degradation of the amniochorion and cervix, are associated with aberrant changes in circulating MMPs, resulting in imbalanced MMP:TIMP ratios and leading to preterm labor. We therefore sought to determine the maternal serum concentrations of MMP-3, MMP-9 and all four TIMPs as well as the MMP:TIMP ratios during term and preterm labor.

## Materials and Methods

### Ethics Statement

The study was approved by the Ethical Committee of Ghent University hospital (EC/2009/010). All participants provided oral and written informed consent.

### Study Design and Population

We performed a prospective cohort study (March 2009 to December 2011) in which 768 pregnant women between 24 and 42 weeks’ gestation, presenting to the labor and delivery ward of Ghent University hospital, a 1000-bed tertiary referral facility, were enrolled in order to build a bank of biological samples and clinical data and to explore putative associations between inflammatory markers of term and preterm labor. All subjects for this study were selected from the prospective cohort, except patients in group 2 (see below). A convenience sample of 166 singleton pregnancies was selected and divided into four groups according to gestational age (GA) and labor status: *Group 1* women with preterm labor (PTL), allocated to the PTB group when delivered before 34 weeks gestation (*PTB*) (n = 47). This group included 32 patients with preterm premature rupture of the membranes (PPROM) and 15 with PTL and intact membranes. *Group 2* consisted of women not in labor, attending the prenatal clinic of Ghent University Hospital and matched for week of gestation with the PTB group. All these women had an uncomplicated pregnancy that proceeded to term delivery (*GA matched controls*) (n = 47). *Group 3* consisted of normal pregnant women at term in labor (*AT in labor*) (n = 40). This group included patients in labor with intact membranes (n = 20) and women with rupture of the membranes (PROM) (n = 20). *Group 4* consisted of healthy pregnant women at term not in labor, undergoing a primary Caesarean section *(AT not in labor*) (n = 32). Because of logistic reasons, MMP-3 analyses were performed on a non-selective sample of 116 singleton pregnancies, divided among the subgroups as follows: 34 *PTB*, 34 *GA matched controls*, 27 *AT in labor* and 21 *AT not in labor*. Inclusion criteria were age ≥18 years, gestational age ≥24 weeks, absence of fetal (congenital) malformations, absence of infectious disease (e.g. HIV, hepatitis B), acute infection and Dutch speaking. Maternal demographic, medical and obstetrical data were collected upon admission.

### Definitions

PTL was defined as having regular uterine contractions (six to twelve contractions in one hour) with cervical changes before 37 completed weeks of gestation. Cervical changes include cervical effacement or dilatation, cervical shortening (<25 mm) and/or funneling and were measured by vaginal examination or transvaginal sonography. PPROM was defined as amniorrhexis before the onset of PTL. A confirmatory test (crystallization test on slide or rapid rupture of membranes (ROM) - test (Amnisure, Boston, US)) was performed if PPROM was suspected on the basis of fluid leakage or oligohydramnion. In case of a positive test, the diagnosis of PPROM was considered. PTB was defined as PTL and/or PPROM that led to a delivery before 34 weeks of gestation. Gestational age was determined based on last menstrual period, corrected by early ultrasound before 20 weeks gestation.

### Sample Collection and Processing

Blood samples of laboring women (either term or preterm) were collected by the attending midwife upon admission to the labor and delivery ward. Women at term not in labor were sampled prior to their Caesarean section. *GA matched controls* were enrolled from the prenatal clinic. These pregnant women were screened at 20–22 weeks (structural ultrasound) to verify whether they fulfilled the inclusion criteria. When they were eligible for participation, the study was explained and they were matched for week of gestation with a PTB case. Sampling was performed during a subsequent prenatal consultation at the appropriate gestational age.

Serum was obtained after coagulation of blood in the presence of clot activator, followed by centrifugation for 10 minutes at 1000 *g* at room temperature and than frozen at minus 80°C until analysis. Samples used for this analysis were never thawed previously. MMP concentrations (MMP-3, -9 and -13) were determined using a Human Matrix Metalloproteinases 3-Plex Panel (Invitrogen, Inc. Carlsbad, CA). The 3-Plex was validated for serum by performing spike and recovery and linearity-of-dilution experiments. MMP-13 concentrations in maternal serum were undetectable using this method in all samples. At a twofold dilution of serum, concentrations were below the detection limit and recovery fell outside the range 70–130%. Recovery and linearity-of-dilution were within the acceptable range for MMP-3 (97–116%) and MMP-9 (74–114%). However, different serum dilutions were required to obtain fluorescence signals within the linear range of detection (an 80-fold dilution for MMP-9 and a 2-fold dilution for MMP-3). TIMP levels (TIMP-1, -2, -3 and -4) were assessed using a Human TIMP Multiplex Kit according to the manufacturers’ instructions (R&D systems, Minneapolis, MN). MMP and TIMP levels were measured using Luminex technology (Luminex 200, Luminex Corporation, Austin, TX).

### Statistical Analysis

Univariate group differences were tested with Fisher’s Exact test for categorical and Mann-Whitney *U*-test for continuous variables. As multiple markers were considered as outcome variables, we accounted for multiple testing by applying the Bonferroni correction: where appropriate, adjusted p-values were obtained by multiplying with the total number of markers and ratios and used to evaluate significance.

The normality of continuous data was evaluated using the Kolmogorov-Smirnov test and visual inspection of QQ-plots. Since the distributions of MMPs and TIMPs were positively skewed, their natural log transformed values were used so as to have normally distributed outcome variables for multiple regression analysis, which was performed on the full dataset. The subgroups were translated into three variables: preterm (vs. at term), labor (vs. not in labor) and rupture of membranes (ROM) (vs. intact membranes).

Because these key covariates were the focus of our investigation, they remained in the models regardless of their significance. To adjust for possible confounding effects, the following covariates were considered in the model selection procedure: maternal age, education level, marital status, smoking, body mass index (BMI), history of PTB, storage time and time delay between sampling and processing (referred to as sample age). This set of covariates was included in the initial model of the selection procedure for each outcome. Model selection was carried out for each outcome independently and occurred in two steps. First, a backward selection of main terms was applied in which covariates were sequentially removed in order of increasing significance until only terms with p-value below 0.10 remained. In the second step, first order interactions were considered between the covariates remaining in the model. The forward selection of interaction terms was performed with an inclusion criterion of p = 0.05. When no further interactions met this criterion, the final model was obtained for that outcome.

Unless noted otherwise, all statistical analyses and tests were performed two-sided at the 5% significance level using SPSS statistics 19 software (IBM, Chicago, Illinois).

## Results

### Demographic and Clinical Characteristics of the Study Population

Maternal and clinical characteristics of the study population (n = 166) are summarized in Table 1. No significant differences were found regarding pre-pregnancy BMI, marital status, ethnicity, conception, parity and history of PTB. Women with *PTB* had a significantly lower education level than *GA matched controls* (P = 0.003). There were significantly more smokers among women with *PTB* as compared to women *AT in labor* (P = 0.007). Significant differences were also found in maternal age (P = 0.03) and the proportion of smokers (P = 0.04) between women *AT not in labor* and women *AT in labor*. As these comparisons evaluated different aspects of the study population and were not part of a family of tests, no correction for multiple testing was applied. Demographic and clinical characteristics were similar for the 116 singleton pregnancies included in the MMP-3 analysis (data not shown).

**Table 1:** Baseline characteristics and obstetric outcome of the study population (n = 166).

	Group 1PTB (n = 47)	Group 2GA matched controls (n = 47)	Group 3AT in labor (n = 40)	Group 4AT not in labor (n = 32)	Group 1 vs. 2P-value	Group 3 vs. 4P-value	Group 1 vs.3P-value
**BASELINE CHARACTERISTICS**							
Maternal age (Me, IQR, y)	29.0 [25.0–33.0]	30.0 [27.0–33.0]	29.0 [27.0–32.0]	32.0 [28.5–35.0]	P = 0.52	P = 0.03	P = 0.83
Pre-pregnancy BMI (Me, IQR, kg/m^2^)	21.5 [19.7–24.9]	21.5 [19.9–23.0]	21.9 [19.9–24.0]	21.6 [19.9–25.0]	P = 0.83	P = 0.43	P = 0.89
Educational level (n,%)					P = 0.003	P = 0.79	P = 0.12
Secondary education or less	21 (44.7)	7 (14.9)	11 (27.5)	7 (21.9)			
Higher education	26 (55.3)	40 (85.1)	29 (72.5)	25 (78.1)			
Marital status (n,%)					P = 1.00	P = 1.00	P = 0.37
Married or cohabiting	43 (91.5)	44 (93.6)	39 (97.5)	31 (96.9)			
Living alone	4 (8.5)	3 (6.4)	1 (2.5)	1 (3.1)			
Smoking at recruitment	8 (17.0)	5 (10.6)	0 (0.0)	4 (12.5)	P = 0.55	P = 0.04	P = 0.007
Ethnicity (n,%)					P = 1.00	P = 0.12	P = 0.18
White/Caucasian	46 (97.9)	45 (95.7)	36 (90.0)	32 (100.0)			
Other	1 (2.1)	2 (4.3)	4 (10.0)	0 (0.0)			
GA at recruitment (Me, IQR, wk)	29.0 [26.0–31.0]	29.0 [26.0–31.0]	40.0 [39.0–40.0]	38.0 [38.0–39.0]	P = 1.00	P<0.001	P<0.001
Conception (n,%)					P = 1.00	P = 0.12	P = 1.00
Spontaneous	39 (83.0)	40 (85.1)	34 (85.0)	31 (96.9)			
Assisted reproductive technology	8 (17.0)	7 (14.9)	6 (15.0)	1 (3.1)			
Nullipara (n,%)	29 (61.7)	22 (46.8)	20 (50.0)	12 (37.5)	P = 0.21	P = 0.34	P = 0.29
History PTB	4 (8.5)	2 (4.3)	2 (5.0)	1 (3.1)	P = 0.68	P = 1.00	P = 0.68
**OBSTETRIC OUTCOME**							
GA at recruitment (Me, IQR, wk)	29.0 [26.0–31.0]	29.0 [26.0–31.0]	40.0 [39.0–40.0]	38.0 [38.0–39.0]	P = 1.00	P<0.001	P<0.001
GA at delivery (Me, IQR, wk)	30.0 [28.0–33.0]	40.0 [39.0–40.0]	40.0 [39.0–40.0]	38.0 [38.0–39.0]	P<0.001	P<0.001	P<0.001
Delivery mode (n,%)					P = 0.49	P<0.001	P = 0.62
Vaginal birth	44 (93.6)	41 (87.2)	39 (97.5)	0 (0.0)			
Caesarean section	3 (6.4)	6 (12.8)	1 (2.5)	32 (100.0)			
Birth weight (Me, IQR, g)	1500.0 [1057.0–1915.0]	3410.0 [3196.3–3832.5]	3450.0 [3212.5–3695.0]	3177.5 [2947.5–3417.5]	P<0.001	P = 0.008	P<0.001
Gender (n,%)					P = 0.04	P = 0.81	P = 0.05
♀	14 (29.8)	24 (52.2)	21 (52.5)	18 (56.3)			
♂	33 (70.2)	22 (47.8)	19 (47.5)	14 (43.8)			

Me, Median; IQR, interquartile range; BMI, body mass index; GA, gestational age; PTB, preterm birth; AT, at term.

Group differences were evaluated with Fisher’s Exact test for categorical variables and Mann-Whitney *U*-test for continuous variables.

### Serum MMPs and TIMPs Concentrations and MMP:TIMP Ratios

MMP-3, TIMP-1, TIMP-2 and TIMP-4 were detectable in all serum samples, MMP-9 in 97.2% and TIMP-3 in only 26.7% of samples. Serum MMP-9 concentrations of 4 samples (all patients from the *PTB* group) were outside the linear range and omitted from further analysis. TIMP-3 levels were below detection limits of the assay in most samples (75.3%) and this parameter was therefore omitted from further analyses. As mentioned previously, MMP-13 could not be assessed in serum. Because we investigated 2 MMPs, 3 TIMPs and 6 MMP:TIMP ratios, we obtained Bonferroni-adjusted p-values by multiplying p-values with a factor 11. In the text, we report the adjusted p-values. Serum MMP-3, and -9, TIMP-1, -2 and -4 concentrations and their ratios are summarized in Table 2.

**Table 2:** Comparison of MMP-3, MMP-9, TIMPs levels and MMP:TIMPs ratios in maternal serum among groups.

	Group 1PTB (n = 47)	Group 2GA matched controls (n = 47)	Group 3AT in labor(n = 40)	Group 4AT not in labor (n = 32)	Group 1 vs.2 *P*-value $	Group 3 vs.4 *P*-value$	Group 1 vs.3 *P*-value$
MMP-9	1125.1 [694.1–1977.4]	639.5 [513.8–924.5]	828.7 [420.7–1259.8]	554.6 [377.5–711.3]	<0.001 (**0.001**)	0.06 (NS)	0.006 (0.07)
TIMP-1	132.04 [118.25–152.27]	125.65 [111.07–139.21]	149.49 [133.01–185.46]	137.98 [125.18–149.44]	0.08 (NS)	0.02 (NS)	0.008 (0.09)
TIMP-2	121.76 [107.62–132.19]	121.26 [109.35–135.82]	156.12 [139.67–190.19]	137.71 [124.57–164.27]	0.68 (NS)	0.03 (NS)	<0.001 (**<0.001**)
TIMP-4	1.46 [1.09–1.96]	1.04 [0.83–1.26]	1.24 [1.12–1.46]	1.08 [0.81–1.35]	<0.001 (**<0.001**)	0.01 (NS)	0.08 (NS)
MMP-9:TIMP-1 ratio	8.21 [4.82–13.88]	5.26 [3.89–7.09]	4.85 [2.86–8.98]	3.94 [3.07–5.33]	0.002 (**0.02**)	0.35 (NS)	<0.001 (**0.006**)
MMP-9:TIMP-2 ratio	9.68 [6.06–14.34]	5.23 [3.95–7.35]	4.61 [2.80–6.75]	3.69 [2.92–5.45]	<0.001 (**<0.001**)	0.26 (NS)	<0.001 (**<0.001**)
MMP-9:TIMP-4 ratio	847.3 [386.8–1463.3]	657.4 [434.0–995.9]	578.0 [342.7–980.3]	567.7 [337.6–765.7]	0.29 (NS)	0.57 (NS)	0.13 (NS)
	PTB (n = 34)	GA matched controls (n = 34)	AT in labor (n = 27)	AT not in labor (n = 21)	*P*-value$	*P*-value$	*P*-value$
MMP-3	9.10 [5.83–15.53]	8.78 [4.68–14.97]	9.55 [3.96–15.83]	7.14 [4.76–13.46]	0.55 (NS)	0.76 (NS)	0.52 (NS)
MMP-3:TIMP-1 ratio	0.065 [0.046–0.111]	0.065 [0.032–0.136]	0.062 [0.033–0.108]	0.057 [0.035–0.093]	0.60 (NS)	0.88 (NS)	0.25 (NS)
MMP-3:TIMP-2 ratio	0.073 [0.058–0.118]	0.074 [0.041–0.111]	0.048 [0.031–0.103]	0.045 [0.030–0.102]	0.47 (NS)	0.99 (NS)	0.03 (NS)
MMP-3:TIMP-4 ratio	6.13 [3.86–12.40]	7.95 [4.84–13.37]	8.45 [3.94–11.37]	7.36 [4.06–11.37]	0.44 (NS)	0.96 (NS)	0.74 (NS)

Results are expressed as median (interquartile range) (ng/ml), group differences were evaluated with the Mann-Whitney *U*-test.

PTB, preterm birth; PT, preterm; AT, at term; NS, not significant.

$ Unadjusted P values and Bonferroni-adjusted P values (adjusted for 11 tests) between brackets.

#### Women with preterm birth vs. GA matched controls

Median levels of MMP-9 and TIMP-4 were significantly higher in women with PTB compared to GA matched controls (respectively P = 0.001 and P<0.001). The same was true for median MMP-9:TIMP-1 and MMP-9:TIMP-2 ratios (respectively P<0.02 and P<0.001).

#### Women at term in labor vs. at term not in labor

No significant differences in MMP-9, MMP-3, all TIMP concentrations or any of the MMP:TIMP ratios were observed between women *AT in labor* vs. *AT not in labor*.

#### Women with preterm birth vs. at term in labor

Median TIMP-2 levels were significantly lower in women with *PTB* compared to those *AT in labor* (P<0.001). A significant higher MMP-9:TIMP-1 and MMP-9:TIMP-2 ratio was observed in women with *PTB* (respectively P = 0.006 and P<0.001). Higher MMP-9 and lower TIMP-1 concentrations were observed in women with *PTB*, but these differences were marginally not significant (respectively P = 0.07 and P = 0.09).

#### Women with PPROM vs. PTL and intact membranes

In the *PTB* group, no significant differences were observed in MMP-9, MMP-3 or any of the MMP:TIMP ratios between women with *PPROM* compared to those with *PTL and intact membranes* (data not shown).

### Determinants of MMP-3, MMP-9, TIMPs and MMP:TIMP Ratios

Multiple regression analysis was performed on the full dataset. After model selection, no significant interaction effects between the covariates were found for any outcome. Results of the final models are shown in Table S1. Since the models used the natural log of marker concentration as outcome variable, model coefficients reflect differences on the ln(concentration) scale. To allow interpretation on the original concentration scale, we also provide exponentiated coefficients that reflect relative (percent wise) instead of absolute changes. The R^2^ of our regression models varied from 7 to 33%, indicating a large amount of variation in log marker concentration (ratio) not explained by the covariates included.

### Preterm vs. at Term

Our regression results showed that, after adjusting for other covariates, MMP-9 and all MMP-9:TIMP ratios were significantly higher for *preterm* (vs. at term, P<0.001), whereas TIMP-1 and TIMP-2 were significantly lower for *preterm* (P = 0.002 resp. P<0.001). In particular, the average MMP-9 concentration as well as all MMP-9:TIMP ratios were more than double *preterm* while the average TIMP-1 and TIMP-2 concentrations were lower (respectively 11% and 22%). Regression results show no significant association between *preterm* and MMP-3 or any of the MMP-3:TIMP ratios.

### Labor vs. No Labor

The regression results showed that, after adjusting for other covariates, MMP-9 concentrations and MMP-9:TIMP-2 ratios were significantly higher for *labor* (vs. no labor, resp. P = 0.03 and P = 0.04), the same was true for TIMP-4 concentrations (P<0.001). In particular, the average MMP-9 concentration and MMP-9:TIMP-2 ratio were 51% resp. 49% higher in *labor*. Additionally, the average TIMP-4 concentration was 33% higher in *labor*. MMP-9:TIMP-1 ratios tended to be higher (±32%) in *labor*, although the adjusted p-value was not significant. Regression results show no significant association between *labor* and MMP-3 or any of the MMP-3:TIMP ratios.

### Rupture of the Membranes vs. Intact Membranes

The regression results show no significant association between *rupture of the membranes* and MMP, TIMP concentrations or any of their ratios.

### Other Covariates

Other important covariates withheld in the regression models are history of PTB, storage time (i.e. window between storage and analysis) and BMI.

Our regression results showed that MMP-9 concentrations and all MMP-9:TIMP ratios tended to be lower for women with a *history of PTB*, but not significantly. For example, the average MMP-9 concentration was 39% lower in women with a *history of PTB*. Furthermore, regression results demonstrated that *storage time* was significantly associated with MMP-9 concentrations and all MMP-9:TIMPs ratios (all P<0.05). With other variables held constant, MMP-9 concentrations multiplied with a factor 1.010 (i.e. increased with 1%) for every additional week of storage. Finally, MMP-3 concentrations and MMP-3:TIMP-1 and MMP-3:TIMP-4 ratios decreased with increasing *body mass index* (all P<0.10).

## Discussion

The role of MMPs and to a lesser extent of TIMPs has been widely investigated in human term and preterm parturition over the past decades. The novelty of our study is the determination of MMP-3, MMP-9 and all four TIMPs in maternal serum. A number of studies have explored MMP-9 in amniotic fluid[11], [19]–[24], [26], [35], [36]. This requires invasive procedures, while blood samples can be easily obtained during pregnancy.

In line with previous observations in maternal plasma [37] and amniotic fluid [22], [24], we found that MMP-9 concentrations were elevated in maternal serum during preterm labor. In contrast, we observed no significant increase in MMP-9 levels during term parturition. Furthermore, our results showed that labor was not associated with a change in serum MMP-3 concentration. To our knowledge, no previous study measured serum MMP-3 during labor, although Park et al [29] reported that MMP-3 levels were elevated in amniotic fluid from women with spontaneous labor at term and preterm.

TIMPs are endogenous specific inhibitors that have been shown to regulate the proteolytic activity of MMPs in normal and pathological processes. A fully functional TIMP network has been demonstrated in human fetal membranes [32], [33], in placenta and decidua [33], in the extra-embryonic coelomic fluid [38] and in amniotic fluid during the second trimester [38]. The four inhibitors were present in maternal serum, but TIMP-3 levels could not be quantified in the majority of the samples.

Multiple regression analysis showed that TIMP-1 and TIMP-2 levels were lower in preterm gestations compared to those at term, irrespective of labor status. This is in agreement with Clark et al [39], who demonstrated that TIMP-1 is suppressed during pregnancy with increasing serum levels from 37 weeks onwards, back to pre-pregnant levels. The similar observation was made in amniotic fluid for TIMP-2 showing an increase in concentration with advancing gestational age [34]. By contrast, amniotic fluid levels of TIMP-1 have been shown to decrease with advancing gestation [24], [26].

TIMP-4 is the most recently identified member of the TIMP family and differs from the other TIMPs in its restricted expression pattern and its structure [40]. Recent reports showed that TIMP-4 is also disregulated during cancer invasion and progression of several organs (for instance in reproductive organs) which highlights its potential role as a biomarker or therapeutic target of disease [40]. Previous research focused on TIMP-4 expression in intrauterine compartments during early and term pregnancy and term parturition. Fortunato et al [32] showed a sparse and inconsistent TIMP-4 expression and low mRNA levels of TIMP-4 in the fetal membranes from laboring and non-laboring women. Furthermore, TIMP-4 was present predominantly in the extra-embryonic coelomic fluid, with low amounts in the amniotic fluid of the first trimester, but significantly increasing with gestation [38]. To the best of our knowledge, there is no information on TIMP-4 in women with preterm labor. Our results showed a significant increase in serum TIMP-4 concentrations during labor (either term or preterm).

Imbalances in the MMP:TIMP ratios may underlie the pathogenesis of various diseases [41], [42] and have been associated with progression [43], remission [44] and severity [45] of disease. Importantly, case-control group comparison showed higher MMP-9:TIMP-1 and MMP-9:TIMP-2 ratios in women with preterm labor. These ratio shifts agreed with the higher MMP-9 and lower TIMP-1 and TIMP-2 concentrations in preterm labor. Multiple regression analyses showed a strong association with preterm status, as MMP-9:TIMP-1 and MMP-9:TIMP-2 ratios were markedly significant higher for the key covariate preterm, while for the key covariate labor only the MMP-9:TIMP-2 ratio was significantly higher. Only few studies have investigated MMP:TIMP ratios during preterm labor. One previous study of Fortunato et al [11] found that the molar ratio between MMP-2 and TIMP-2, but not TIMP-1 was increased in amniotic fluid during PPROM.

None of the MMPs or TIMPs concentrations and MMP:TIMP ratios differed significantly between women with PPROM and those with PTL and intact membranes. This finding is consistent with Romero et al [25] who found higher MMP-9 concentrations in the fetal compartments, but not in maternal plasma [25]. These observations suggest that cytokine changes during pregnancy are not always reflected in the maternal circulation which is in line with data from animal models indicating that that alterations in cytokine profiles are strictly compartmentalized and independently regulated [46]. Recently, Brou et al [47] evaluated thirty-six biomarkers in maternal, fetal and intra-amniotic compartment of women with preterm labor and demonstrated that the biomarkers of the PTB pathway differ between different compartments.

It has been shown that pre-analytical variability arising from sampling and storage procedures affect assayed concentration of biological markers [48], [49]. The impact of storage time and sample age on MMP and TIMP concentrations was considered in the multiple regression analysis. Our regression results showed that storage time was positively correlated with serum MMP-9 concentration after adjusting for other covariates. Studies investigating MMP-9 stability during long-term storage at −80°C show conflicting results [50], [51]. The reason for these discrepant results is not clear. In our study, we found no interactions between storage time and other covariates in the model, indicating no differences in storage time between groups.

Some limitations of this study deserve consideration. First, the relatively small sample size of this study. More women with PPROM and PTL and intact membranes might be necessary to determine differences in MMPs and TIMPs concentrations. Therefore, large studies are required to confirm our results. Since we conducted a case control study and MMP and TIMP concentrations were measured only once upon admission, we were unable to demonstrate the exact timing of the ratio shift between MMP and TIMP. It has been shown that MMPs are temporally regulated to perform specific functions during pregnancy [8]. For example, MMP-9 is selectively expressed in the decidua and fetal membranes with the onset of labor, but barely detectable before labor [4]. Furthermore, it would be interesting to evaluate whether MMP:TIMP ratios differ between patients with preterm labor who delivered preterm and those delivering at term. A preliminary evaluation showed no significant differences, but the number of patients with preterm labor and term delivery was rather low (n = 10). Finally, we only evaluated immunoreactive forms of MMP-3 and -9 with a MMP 3–plex detecting all circulating MMPs (including pro, active and TIMP bound forms). An alternative would be to evaluate the enzymatic activity of MMPs, because the mere presence of MMP does not necessarily establish their catalytic capacity [8]. Fortunato et al [11] demonstrated that only a small amount of total amniotic fluid MMP-9 and -2 were active. However, it has been shown that the immunoreactivity of MMP-9 in amniotic fluid correlates well with its enzymatic activity [23].

In conclusion, this study showed that MMP-9:TIMP-1 and MMP-9:TIMP-2 balances in maternal serum are tilting in favor of gelatinolysis in women with preterm labor. All four TIMPs were expressed in maternal serum. While TIMP-1 and -2 concentrations were lower during gestation, irrespective of labor, TIMP-4 levels were elevated during labor (either at term or preterm). The observations in the present study indicate that circulating MMPs and TIMPs may also play a role in the pathogenesis of preterm labor at systemic level or at least reflect pregnancy and labor status. Our findings provide the possibility to develop a far less invasive approach (compared to amniocentesis) for measurement of enzymes essential for ECM remodeling during pregnancy and parturition. However, a great deal still remains to be learned about MMPs and in particular TIMPs during various stages of normal pregnancy and labor as well as in pathological conditions such as preterm labor.

## Supporting Information

Table S1 **Multiple regression model for ln(MMP-9, MMP-3, TIMPs levels and MMP:TIMP ratios.** This is a table in PDF format. The file can be viewed with Adobe Acrobat reader
(DOCX)
