# Supplementary material for: Imbalances between Matrix Metalloproteinases (MMPs) and Tissue Inhibitor of Metalloproteinases (TIMPs) in Maternal Serum during Preterm Labor
Source: PLoS One. 2012 Nov 8;7(11):e49042. doi: 10.1371/journal.pone.0049042 (PMC3493509; doi:10.1371/journal.pone.0049042)
Supplement: Table S1 — Multiple regression model for ln(MMP-9, MMP-3, TIMPs levels and MMP:TIMP ratios. This is a table in PDF format. The file can be viewed with Adobe Acrobat reader (DOCX) [file pone.0049042.s001.docx]

Table S1: Multiple regression model for ln(MMP-9, MMP-3, TIMPs levels and MMP:TIMP ratios)

| **Outcome**  **variable** | **n** | **Parameter** | **Model coefficient**  **[95%CI]** | **Exponentiated coefficient [95%CI]** | **Unadjusted**  ***P*-value** | **Adjusted**  ***P*-value**^$^ | ***R²*** |
| --- | --- | --- | --- | --- | --- | --- | --- |
| MMP-9 | 162 | Intercept | 5.575 [5.138, 6.012] | 263.7 [170.4, 408.3] | <0.001 | <0.001 | 0.23 |
|  |  | Preterm [vs. at term] | 0.794 [0.466, 1.122] | 2.212 [1.594, 3.071] | <0.001 | **<0.001** |  |
|  |  | Labor [vs. not in labor] | 0.411 [0.147, 0.675] | 1.508 [1.158, 1.964] | 0.002 | **0.03** |  |
|  |  | ROM [vs. intact membranes] | -0.188 [-0.453, 0.078] | 0.829 [0.636, 1.081] | 0.17 | NS |  |
|  |  | History of PTB [vs. no history] | -0.489 [-0.898, -0.080] | 0.613 [0.407, 0.923] | 0.02 | NS |  |
|  |  | Storage time (in weeks) | 0.010 [0.004, 0.017] | 1.010 [1.004, 1.017] | 0.001 | **0.01** |  |
| TIMP-1 | 166 | Intercept | 4.761 [4.563, 4.959] | 116.9 [95.9, 142.5] | <0.001 | <0.001 | 0.15 |
|  |  | Preterm [vs. at term] | -0.113 [-0.174, -0.053] | 0.893 [0.840, 0.948] | <0.001 | **0.003** |  |
|  |  | Labor [vs. not in labor] | 0.078 [-0.001, 0.156] | 1.081 [0.999, 1.169] | 0.05 | NS |  |
|  |  | ROM [vs. intact membranes] | 0.028 [-0.056, 0.112] | 1.028 [0.946, 1.119] | 0.51 | NS |  |
|  |  | BMI (kg/m²) | 0.008 [-0.001, 0.016] | 1.008 [0.999, 1.016] | 0.08 | NS |  |
| TIMP-2 | 166 | Intercept | 5.275 [5.064, 5.486] | 195.4 [158.2, 241.3] | <0.001 | <0.001 | 0.33 |
|  |  | Preterm [vs. at term] | -0.244 [-0.309, -0.179] | 0.783 [0.734, 0.836] | <0.001 | **<0.001** |  |
|  |  | Labor [vs. not in labor] | 0.006 [-0.077, 0.089] | 1.006 [0.926, 1.093] | 0.89 | NS |  |
|  |  | ROM [vs. intact membranes] | 0.053 [-0.036, 0.142] | 1.054 [0.965, 1.153] | 0.24 | NS |  |
|  |  | Smoker [vs. no smoker] | -0.140 [-0.245, -0.035] | 0.869 [0.783, 0.966] | 0.009 | 0.10 |  |
|  |  | BMI (kg/m²) | -0.011 [-0.020, -0.002] | 0.989 [0.980, 0.998] | 0.02 | NS |  |
| TIMP-4 | 166 | Intercept | -0.007 [-0.101, 0.088] | 0.993 [0.904, 1.092] | 0.89 | NS | 0.16 |
|  |  | Preterm [vs. at term] | 0.072 [-0.030, 0.174] | 1.075 [0.970, 1.190] | 0.17 | NS |  |
|  |  | Labor [vs. not in labor] | 0.287 [0.155, 0.420] | 1.332 [1.168, 1.522] | <0.001 | **<0.001** |  |
|  |  | ROM [vs. intact membranes] | -0.010 [-0.153, 0.132] | 0.990 [0.858, 1.141] | 0.89 | NS |  |
| MMP-9:TIMP-1 | 162 | Intercept | 0.645 [0.176, 1.114] | 1.906 [1.192, 3.047] | 0.007 | 0.08 | 0.21 |
|  |  | Preterm [vs. at term] | 0.911 [0.559, 1.264] | 2.487 [1.749, 3.540] | <0.001 | **<0.001** |  |
|  |  | Labor [vs. not in labor] | 0.322 [0.039, 0.606] | 1.380 [1.040, 1.062] | 0.03 | NS |  |
|  |  | ROM [vs. intact membranes] | -0.205 [-0.491, 0.080] | 0.815 [0.612, 1.083] | 0.16 | NS |  |
|  |  | History of PTB [vs. no history] | -0.484 [-0.923, -0.044] | 0.616 [0.397, 0.957] | 0.03 | NS |  |
|  |  | Storage time (in weeks) | 0.010 [0.004, 0.017] | 1.010 [1.004, 1.017] | 0.003 | **0.03** |  |
| MMP-9:TIMP-2 | 162 | Intercept | 0.571 [0.129, 1.012] | 1.770 [1.138, 2.751] | 0.01 | NS | 0.29 |
|  |  | Preterm [vs. at term] | 1.036 [0.704, 1.368] | 2.818 [2.022, 3.927] | <0.001 | **<0.001** |  |
|  |  | Labor [vs. not in labor] | 0.397 [0.130, 0.664] | 1.487 [1.139, 1.943] | 0.004 | **0.04** |  |
|  |  | ROM [vs. intact membranes] | -0.231 [-0.500, 0.037] | 0.794 [0.607, 1.038] | 0.09 | NS |  |
|  |  | History of PTB [vs. no history] | -0.413 [-0.827, 0.001] | 0.662 [0.424, 1.001] | 0.05 | NS |  |
|  |  | Storage time (in weeks) | 0.010 [0.004, 0.016] | 1.010 [1.004, 1.016] | 0.002 | **0.02** |  |

| **Outcome**  **variable** | **n** | **Parameter** | **Model coefficient**  **[95%CI]** | **Exponentiated coefficient [95%CI]** | **Unadjusted**  ***P*-value** | **Adjusted**  ***P*-value**^$^ | ***R²*** |
| --- | --- | --- | --- | --- | --- | --- | --- |
| MMP-9:TIMP-4 | 162 | Intercept | 5.543 [5.061, 6.024] | 255.4 [157.7, 413.2] | <0.001 | <0.001 | 0.13 |
|  |  | Preterm [vs. at term] | 0.760 [0.398, 1.122] | 2.138 [1.489, 3.071] | <0.001 | **<0.001** |  |
|  |  | Labor [vs. not in labor] | 0.120[-0.170, 0.411] | 1.127 [0.844, 1.508] | 0.42 | NS |  |
|  |  | ROM [vs. intact membranes] | -0.192 [-0.485, 0.101] | 0.825 [0.616, 1.106] | 0.20 | NS |  |
|  |  | History of PTB [vs. no history] | -0.634 [-1.084, -0.183] | 0.530 [0.338, 0.833] | 0.006 | 0.08 |  |
|  |  | Storage time (in weeks) | 0.011 [0.004, 0.018] | 1.011 [1.004, 1.018] | 0.002 | **0.02** |  |
| MMP-3 | 116 | Intercept | 3.103 [2.254, 3.951] | 22.27 [9.526, 51.99] | <0.001 | <0.001 | 0.09 |
|  |  | Preterm [vs. at term] | 0.023 [-0.255, 0.300] | 1.023 [0.775, 1.350] | 0.87 | NS |  |
|  |  | Labor [vs. not in labor] | -0.036 [-0.395, 0.323] | 0.965 [0.674, 1.381] | 0.84 | NS |  |
|  |  | ROM [vs. intact membranes] | 0.087 [-0.286, 0.460] | 1.091 [0.751, 1.584] | 0.64 | NS |  |
|  |  | Sample age (in hours) | 0.010 [-0.002, 0.021] | 1.010 [0.998, 1.021] | 0.09 | NS |  |
|  |  | BMI (kg/m²) | -0.049 [-0.085, -0.013] | 0.952 [0.919, 0.987] | 0.008 | 0.08 |  |
| MMP-3:TIMP-1 | 116 | Intercept | -1.762 [-2.677, -0.847] | 0.172 [0.069, 0.429] | <0.001 | 0.002 | 0.08 |
|  |  | Preterm [vs. at term] | 0.184 [-0.112, 0.479] | 1.202 [0.894, 1.614] | 0.22 | NS |  |
|  |  | Labor [vs. not in labor] | -0.004 [-0.377, 0.368] | 0.996 [0.686, 1.445] | 0.98 | NS |  |
|  |  | ROM [vs. intact membranes] | 0.084 [-0.320, 0.487] | 1.088 [0.726, 1.627] | 0.68 | NS |  |
|  |  | BMI (kg/m²) | -0.053 [-0.092, -0.015] | 0.948 [0.912, 0.985] | 0.007 | 0.08 |  |
| MMP-3:TIMP-2 | 116 | Intercept | -2.301 [-3.181, -1.421] | 0.100 [0.042, 0.241] | <0.001 | <0.001 | 0.09 |
|  |  | Preterm [vs. at term] | 0.354 [0.070, 0.638] | 1.424 [1.073, 1.893] | 0.02 | NS |  |
|  |  | Labor [vs. not in labor] | 0.071 [-0.287, 0.429] | 1.073 [0.751, 1.536] | 0.70 | NS |  |
|  |  | ROM [vs. intact membranes] | 0.020 [-0.368, 0.408] | 1.020 [0.692, 1.503] | 0.92 | NS |  |
|  |  | BMI (kg/m²) | -0.034 [-0.071, 0.004] | 0.967 [0.931, 1.004] | 0.08 | NS |  |
| MMP-3:TIMP-4 | 116 | Intercept | 3.120 [2.213, 4.026] | 22.65 [9.143, 56.04] | <0.001 | <0.001 | 0.07 |
|  |  | Preterm [vs. at term] | -0.007 [-0.300, 0.286] | 0.993 [0.741, 1.331] | 0.96 | NS |  |
|  |  | Labor [vs. not in labor] | -0.156 [-0.525, 0.214] | 0.856 [0.592, 1.239] | 0.41 | NS |  |
|  |  | ROM [vs. intact membranes] | 0.107 [-0.293, 0.507] | 1.113 [0.746, 1.660] | 0.60 | NS |  |
|  |  | BMI (kg/m²) | -0.053 [-0.091, -0.014] | 0.948 [0.913, 0.986] | 0.008 | 0.08 |  |

Results of the model fitted on the full dataset , obtained from the model selection procedure outlined in the text. Coefficients of the model (additive on the log scale) were exponentiated to multiplicative factors, allowing interpretation on the concentration scale.

CI, confidence interval; R², R square; ROM, rupture of the membranes; PTB, preterm birth; BMI, body mass index; NS, not significant

$ Bonferroni-adjusted P values (adjusted for 11 tests)
